# Responsible conduct of research: Preparedness for times of crisis

**DOI:** 10.1017/cts.2023.13

**Published:** 2023-03-08

**Authors:** Cynthia Hahn, Emmelyn Kim, Arinayo O. Apara, Tina Chuck, Hallie Kassan, Bettie M. Steinberg

**Affiliations:** 1 Integrated Research Strategy, LLC, Raleigh, NC, USA; 2 The Feinstein Institutes for Medical Research, Northwell Health, Manhasset, NY, USA; 3 The Feinstein Institutes for Medical Research, Northwell Health and Donald and Barbara Zucker School of Medicine at Hofstra/Northwell, Hempstead, NY, USA

**Keywords:** Clinical research in a pandemic, research integrity, trust in science and medicine

## Abstract

A live, virtual conference, “Driving Responsible Conduct of Research during a Pandemic,” was held in April 2021, 13 months after the COVID-19 pandemic fundamentally altered the conduct of clinical research across the USA. New York was an early epicenter of the US pandemic, highlighting preexisting problems in clinical research and allowing us to assess lessons learned and to identify best practices for the future. Risks and opportunities were categorized broadly into three areas, protecting the welfare and safety of human subjects, ensuring trust in science and medicine, and implementing efficient, ethical, and compliant clinical research. Analysis of conference proceedings, and recent publications, shows a need for preparedness that is more effective, robust partnerships, and organizational systems and standards to strengthen the ethical and responsible conduct of research.

## Introduction

The Feinstein Institutes for Medical Research at Northwell Health, in partnership with other health systems, academic medical centers, and healthcare-related organizations in the greater New York metropolitan area, held a live, virtual, interactive conference entitled “Driving Responsible Conduct of Research during a Pandemic” in April 2021, 13 months after COVID-19 fundamentally altered the conduct of clinical research across the USA. New York, an early epicenter of the pandemic in the USA, provided an opportunity to assess lessons learned and identify best practices specifically related to clinical research for potential future events. Conducting clinical research during a pandemic or other public health emergency, especially where no approved treatment or vaccine exists, presents challenges for responsible conduct of research (RCR). For this conference report, we use the National Institutes of Health definition of RCR, “the practice of scientific investigation with integrity” [1], specifically as it applies to the aspects of clinical research discussed below.

Research has shown that systems are ill-equipped to address ethical issues in research during a health emergency [2,3], which were borne out with the COVID-19 pandemic. According to a study published in the *American Journal of Public Health* [4], there is a need to further develop areas in research that adequately address gaps in training, information sharing, and communication to the public, as well as the development of criteria and metrics. Failure to proactively address these gaps puts healthcare organizations and science at risk from losing the public’s trust and more importantly, jeopardizes patient safety. It was with this concern in mind that more than 150 clinicians, scientists, regulators, and administrators gathered virtually to discuss the research community’s experience in battling COVID-19, assess early lessons learned, and identify best practices. Analysis of conference proceedings revealed critical lessons learned and future considerations for the ethical and responsible conduct of clinical research. These findings are discussed below.

The full recording of the conference is available at Feinstein.northwell.edu/RCR.

## Conference Results and Discussion

Many of the problems that were identified and discussed below were not new or unique to the pandemic, but the pandemic put a spotlight on them. Prior planning would have reduced risks to patients and to the research enterprise. Heightened awareness of these structural vulnerabilities will lead to better, more equitable research in the future.

Risks and opportunities identified throughout the conference that related to the responsible conduct and integrity of ongoing and newly proposed clinical research during the pandemic were categorized broadly into three thematic areas:

- Protecting the welfare and safety of human subjects
- Ensuring trust in science and medicine
- Implementing efficient, ethical, and compliant clinical research.

### Theme 1. Protecting the Rights, Welfare, and Safety of Human Subjects

When there are no approved therapeutics to address an outbreak, the healthcare environment will transform by necessity into a research-focused setting. Conference speakers and workshop attendees identified three major vulnerabilities: 1) exclusion of individuals or communities who might otherwise benefit from access to experimental therapeutics, contributing to health inequities; 2) exposing individuals or communities to unproven or ineffective therapeutics; and 3) maintaining operations for current ongoing research, unrelated to the pandemic.

Multiple factors were identified by conference attendees that contributed to these risks. First was the acuity of the pandemic itself. Risks were unknown, changing rapidly, and were local, national, and global issues. People were isolated, making collaboration initially more difficult and often disrupting or altering ongoing studies [5]. Resources to conduct both COVID-19 and non-COVID-19 research were limited due to researchers being pulled into clinical duties and/or becoming ill. Moreover, non-COVID-19 research was administratively halted or significantly altered in many cases. While these responses were necessary, they put studies at risk when not done carefully.

Trial design and methodology for COVID-19 trials were a second factor identified [6]. Mortality was mounting, leading to an urgent need for novel therapies to move from the bench to the bedside. Some trials were not rigorously designed to ensure useful data and endpoints, had difficulty recruiting (due to a placebo or randomization to an unpopular arm), were too complicated, or may not have been adequately powered to be successful.

Operational issues were met with researchers expecting a range of flexibility in regulations that would make research no longer compliant. The shortage of resources for trial conduct and the need to recruit and implement on a large scale contributed to the risks. There was often scarcity of existing collaborations and partnerships within the scientific community and between the scientific community and the public that could be leveraged.

*Another factor identified was the lack of adequate research training for clinical providers and staff. Physicians and nurses were expected to act as clinical scientists, but they may not have had any research training. Standard human subject protection training requirements may be administratively onerous or not tailored for rapid activation. Finally, lack of effective community engagement was a major factor discussed. Historical mistrust of the medical community requires focused attention on structural inequities and thoughtful integration into research design for more effective partnerships* [7].

After the conference, the Secretary’s Advisory Committee on Human Research Protections (SACHRP) published recommendations, “Consideration of the Principle of Justice under 45 CFR part 46,” on July 22, 2021 [8]. They noted that to achieve full equity, Institutional Review Board cannot do it alone, it must be a collaborative effort among sponsors, investigators, the community, and regulators. The conference endorsed this position as well, emphasizing that equity does not occur in a vacuum and is not a single responsibility; it involves cross-community actions and collaborations to be successful. The SACHRP recommendations mirror many of the discussions held during the conference relating to equity, including (1) modernizing and extending the recommendations of the Belmont Report; (2) federal agencies developing formal guidance or regulation that bolsters ethical principles with force; and (3) convening a national conversation that involves the research community, the public (as actual and potential research participants and as the funders of research), and traditionally underserved or excluded communities to develop an extended Belmont Report and guidance or regulations [8].

Another recommendation made by conference attendees was to expand operational clinical research and research integrity training in medical school and allied health curricula and expose medical trainees to the basics of clinical research when conducting clinical rotations. Specifically, they recommended creating training requirements for “non-research” clinicians engaged in the implementation of research, differentiating it from training for researchers and those engaged in the oversight and administration of the research. This training should be simple, accessible remotely, focused on data and research integrity and the safety of participants, and appropriate for adult learners. Recent recommendations by the National Academy of Medicine include policies to support effective funding and resourcing during emergencies and supporting a pipeline of researchers and programs for emergent settings [9].

### Theme 2. Ensuring Trust in Science and Medicine

After COVID-19 was declared an international public health concern in January of 2020, a review of publications in PubMed reported that a large quantity of research publications was processed within a brief period [10]. Rapid publication efforts can help to disseminate critical information during pandemics but also raise concerns about the quality of the information and data integrity, as well as the potential for dissemination of false information that could have far-reaching implications [10,11]. At the time of the conference, Retraction Watch had identified 118 papers related to COVID-19 that were retracted by different journals, citing assorted reasons [12]. A later study reported that papers dealing with COVID-19 were accepted 11.5 times faster than for influenza in 2020, suggesting a rush to publish and a lack of quality control in scientific publishing and the peer review process [13]. Most retractions with identified reasons were for ethics violations, with 30% from one group of authors. A second study, comparing retractions of papers about COVID-19 to all medically related retracted papers between February 2020 and May 2022, found that papers dealing with COVID-19 were retracted more quickly after publication and many were pre-prints, suggesting a higher level of scrutiny [14]. Most appeared to be retracted for non-ethical reasons, but a large fraction was retracted with no explanation given. Future studies will be needed to determine whether ethical misconduct was more common with COVID-19 publications.

Trust in science and medicine is a political hot topic and one of the many challenges faced by the medical community, due in part to lack of trust in the information individuals and communities receive through traditional and non-traditional media [15]. Transparency and information sharing was a key concept discussed throughout the conference. As attendees discussed, the COVID-19 pandemic could not have hit at a worse time in American political discourse with the country so divided. Conference moderators and panelists were careful to balance any discussion of the national climate with discussions of what the scientific community can control, specifically data generation, management, analysis, and publication. It was noted repeatedly that it is difficult to correct misinformation once it is made public. This is the case for the public and for scientists and clinicians. Scientific publications related to COVID-19 were being referenced 2 years after they had been retracted [13].

Specific risks and vulnerabilities identified by speakers and attendees included a lack of scientific and health literacy in the general population, medical community, politicians, media, and regulators. This contributed to miscommunication or misunderstanding of study data or results, or when new or contradictory evidence appeared. Updating information in the scientific literature, public press, and social media was difficult. Misaligned goals and expectations for clinical trials and their outcomes amongst scientists, clinicians, and the public led to mistrust and therapeutic misconceptions. Finally, the medical and scientific communities needed to effectively guard against pressures such as investigators over-promising results without sufficient evidence. A recent study on misinformation about COVID-19 found that individuals with greater trust in science were more skeptical about misinformation, supporting the notion that greater levels of trust can be protective [16].

The medical and scientific communities should evaluate the roles they play in the dissemination of information to the public and in mitigating therapeutic misconceptions. The process for issuing retractions needs to be further evaluated, and a standardized system is needed to identify retractions across journals. Careful dissemination practices, including publishing negative studies, need to focus on higher quality evidence due to risks of actions taken by the public and policy impacts from lower quality evidence [9,17]. While the problems discussed above were not unique to the pandemic, they were clearly highlighted during a vulnerable period.

### Theme 3. Implementing Efficient, Ethical, and Compliant Clinical Trials

COVID-19 exposed weaknesses in the existing clinical trials system or accelerated trends, serving as a real-world laboratory for evaluating changes in the operations of clinical trial implementation. Specific risks and vulnerabilities identified during the conference included rapid adoption of changes in the business model and implementation of regulatory flexibility in the conduct of clinical trials without adequate review, monitoring, and evaluation of those changes. These included changes in roles and responsibilities, increased adoption of decentralized clinical trial models without careful consideration or management of associated risks, and increased adoption of remote communication and digital health technology to support clinical trials with its attendant privacy and security risks [18]. Lack of access to technology or the ability or knowledge to use the technology by certain communities can create further inequities. These factors could lead to increased risk to participants, increased liability for sponsors and investigative sites, and/or data and research integrity issues.

To address these risks, conference attendees recommended that organizations conduct risk assessments on research protocols based on certain criteria. Protocols with new investigators, vulnerable populations, complicated assessments, etc. could be assigned a higher risk score and prioritized when conducting routine auditing and monitoring. Also, organizations should ensure that there are gatekeepers to confirm that the necessary approvals and institutional requirements are met prior to conducting research, which will require collaboration and support from organization leadership on all levels.

Conference attendees also discussed evaluating the flexibility and guidance provided by regulators during the pandemic. The regulatory flexibility employed in certain sectors during COVID-19 (research, healthcare privacy, reimbursement, licensure/privileges, informed consent via telemedicine, etc.) should be individually evaluated for effectiveness to inform for potential permanent changes in regulation or guidance. The patchwork of state laws related to data ownership and security is challenging and even more so during emergencies and pandemics. Therefore, greater coordination amongst state laws should be pursued. Assessing and implementing guidance to address the use of digital health technology are needed to support more effective data collection and dissemination while ensuring privacy protections and data security in clinical research. Establishing and mandating clear requirements to accelerate data sharing, independent of funding source, during an emergency or pandemic while maintaining rigor and reproducibility were highlighted in another report [9].

Recommendations discussed for healthcare institutions include improving funding resources to support the conduct, oversight, and implementation of clinical trials in non-traditional settings. Interdisciplinary teams including non-research members in healthcare settings are critical to successful implementation. Attendees recommended that teams be established prior to a specific need and well-coordinated at sites for rapid adoption and implementation. There should also be incentives and mechanisms to address responsible data sharing and collaboration.

Strategies to better engage community partners in research were discussed, specifically through implementing effective and intentional community engagement and developing sustainable and effective community networks before the next pandemic. This is because advancing health equity requires elevation and empowerment of the community to inform prioritization of research efforts in partnership with private and public organizations. Community engagement during pandemics and at all other times requires broader involvement, so interventions must address structural inequities including social, economic, and health conditions that can pose challenges to research [9,19]. Effective bi-directional communication strategies that are inclusive of linguistic and cultural components while addressing scientific and health literacy can reinforce the intrinsic value of participation and maintain engagement [20].

## Summary of Recommendations

Each identified conference theme provided recommendations for the ethical and responsible conduct of clinical research in a pandemic as well as in general. Enhancing the welfare and safety of human subjects will require addressing the root cause of identified risk including lack of preparedness or planning and weak organizational systems. This includes proactive resourcing and research training of clinical providers, designing more rigorous trials (i.e., quality by design) to ensure useful data and endpoints, and better leveraging collaborations and partnerships to conduct large-scale research. Conference attendees recommended that organizations consider developing a set of standards for conducting research during a crisis. Private–public partnerships focused on implementing clinical trial standards such as the Clinical Trials Transformation Initiative or the Multi-Regional Clinical Trials center could help address these concerns.

Ensuring trust in science and medicine will require more effective community engagement by organizations and greater efforts to increase science and health literacy in the public and media by multiple stakeholders. Standards for generating higher quality evidence, while reducing the potential for confirmation bias in clinical trials, and systems to issue retractions and identify retracted papers in journals are also critical.

Implementing efficient, ethical and compliant clinical trials requires adequate funding, oversight, and a culture of integrity at research organizations. Conference attendees recommended fostering a culture of integrity using a risk-based approach, focusing on high-risk areas when conducting clinical research in emergent situations. Evaluating effectiveness of regulatory flexibility for future implementation is necessary given the changing landscape of research. Interdisciplinary teams need to be established in advance and well-coordinated for rapid implementation. Finally, establishing effective community partnerships and networks to address priorities and structural inequities will better prepare us in advance of the next pandemic.
